# Leaf Venation Architecture in Relation to Leaf Size Across Leaf Habits and Vein Types in Subtropical Woody Plants

**DOI:** 10.3389/fpls.2022.873036

**Published:** 2022-05-06

**Authors:** Guoquan Peng, Yingjie Xiong, Mengqi Yin, Xiaolin Wang, Wei Zhou, Zhenfeng Cheng, Yong-Jiang Zhang, Dongmei Yang

**Affiliations:** ^1^College of Chemistry and Life Sciences, Zhejiang Normal University, Jinhua, China; ^2^School of Biology and Ecology, University of Maine, Orono, ME, United States

**Keywords:** leaf habit, leaf size, leaf vein type, scaling relationship, subtropical forest, vein density, vein distribution

## Abstract

Leaves are enormously diverse in their size and venation architecture, both of which are core determinants of plant adaptation to environments. Leaf size is an important determinant of leaf function and ecological strategy, while leaf venation, the main structure for support and transport, determines the growth, development, and performance of a leaf. The scaling relationship between venation architecture and leaf size has been explored, but the relationship within a community and its potential variations among species with different vein types and leaf habits have not been investigated. Here, we measured vein traits and leaf size across 39 broad-leaved woody species within a subtropical forest community in China and analyzed the scaling relationship using ordinary least squares and standard major axis method. Then, we compared our results with the global dataset. The major vein density, and the ratio of major (1° and 2°) to minor (3° and higher) vein density both geometrically declined with leaf size across different vein types and leaf habits. Further, palmate-veined species have higher major vein density and a higher ratio of major to minor vein density at the given leaf size than pinnate-veined species, while evergreen and deciduous species showed no difference. These robust trends were confirmed by reanalyzing the global dataset using the same major vein classification as ours. We also found a tradeoff between the cell wall mass per vein length of the major vein and the major vein density. These vein scaling relationships have important implications on the optimization of leaf size, niche differentiation of coexisting species, plant drought tolerance, and species distribution.

## Introduction

The leaf is the main organ of photosynthesis in higher plants and a critical component in the plant water transport system, which accounts for 30% or more of whole-plant hydraulic resistance (Sack and Holbrook, 2006). Leaf size is an important determinant of plant physiological function and ecological strategy. It reflects the efficiency of light interception and the ability of carbon capture in plants (Parkhurst and Loucks, 1972; Givnish and Vermeij, 1976). Leaf size also shapes the tradeoff between carbon assimilation and water use efficiency, which is crucial for leaf temperature regulation under different climatic conditions (Michaletz et al., 2014; Fauset et al., 2018; Li et al., 2020). Leaf venation is the main structure for physical support and water/nutrient transport in the leaf, which has an important role in maintaining the growth and development of a leaf. It also transports photosynthate and signal molecules from the mesophyll to the rest of the plant. Thus, leaf venation is strongly related to the leaf hydraulic conductance, gas exchange rates, and plant performance (Niklas, 1999; Sack and Holbrook, 2006; Sack et al., 2012, 2013). The leaf hydraulic conductance (*K*_leaf_) is determined by the conductance of a series of the xylem (*K*_x_) and outside-xylem pathways (*K*_ox_). The vein density is a determinant of both *K*_x_ and *K*_ox_ because higher densities provide more numerous xylem flow pathways that are parallel per leaf area and shorten pathways for water movement outside the xylem (Cochard et al., 2004; Sack and Frole, 2006; Brodribb et al., 2007; McKown et al., 2010). The higher vein densities and conductivities are expected to be adaptations to higher-resource conditions (Sack et al., 2005; McKown et al., 2010), and large leaves are predominant in moister and/or shaded habitats (Givnish, 1987; Fonseca et al., 2000). Smaller leaves and higher major vein densities are more frequent in dry habitats (Givnish, 1987; Ackerly, 2004; Scoffoni et al., 2011). In addition, the development of the algorithm for vein formation during leaf expansion also provides the basis correlation for vein trait and leaf size (Sack and Scoffoni, 2013). Hence, leaf venation has an important role in the optimization of leaf size. In addition, the variation of leaf size would be closely related to leaf venation architecture.

The scaling of vein traits with leaf size across species has been explored by several studies, but not systematically (except for Sack et al., 2012, 2013). Hence, the conclusions are not consistent. Previous studies with fewer species (≤10) found a negative correlation between major vein density and leaf size, while no relationship between minor vein density and leaf size was found in most studies (Sack et al., 2008; Dunbar-Co et al., 2009; Scoffoni et al., 2011). However, Walls (2011) showed a weak relationship (*R*^2^ = 0.11). Then, Price et al. (2012) showed that vein density was independent of leaf size by using an automated analysis of low-resolution images, but did not distinguish vein orders in 339 species collected from the National Cleared Leaf Collection at the Museum of Natural History, Smithsonian Institution. Another study analyzed 485 globally distributed species with new, high-resolution measurements of vein systems (Sack et al., 2012). They found that larger leaves had major veins with larger diameters, but lower major vein density. Meanwhile, minor vein traits were independent of leaf size, and total leaf vein density was not related to leaf size for both palmate-veined (multiple 1° veins) and pinnate-veined (with a single 1° veins) species. These inconsistent conclusions were not only because of the different image resolutions of leaf venation, but also the different classification standards of major veins and minor veins. In Sack et al. (2012), the major vein included 1°, 2°, and 3° veins, while the minor veins were defined as all more higher-order veins (Sack et al., 2012). In contrast, Price et al. (2012) did not distinguish vein orders. However, the major vein might also be defined based on different formation timing and the gene expression during development (Haritatos et al., 2000; Evert, 2006). Based on the newly developed synthetic model for the development of vein hierarchy, the formation of 1° and 2° veins coincides with the first slow phases, while most 3° and higher-order veins form during the rapid expansion phase (Sack et al., 2012). Hence, it could be reasonable to define the major vein as the sum of 1° and 2° veins, and the minor veins as the 3° and higher-order veins. This classification is the same as in Ellis et al. (2009) and Walls (2011), which will be used in this study. To the best of our knowledge, although the scaling relationship between leaf venation architecture and leaf size has been studied within the given genera and families and at the global level, it has not been investigated within a community sharing similar environmental conditions. It is not clear if this general scaling relationship is conserved across species that coexist in a community.

The major and minor veins differ in the timing of development and xylem and phloem formation (Sack et al., 2012; Sack and Scoffoni, 2013). The variation in the ratio of major to minor vein density could affect leaf hydraulic conductance and vulnerability to cavitation (Scoffoni et al., 2011). Hence, the ratio of major to minor vein density should be considered because even within species with the same total vein density and leaf size, leaf vein distribution could be different. Additionally, the major vein continually thickens with the increase of the leaf size, while the diameter of the minor vein quickly reaches the maximum and is kept constant with the further increase in leaf size (Sack et al., 2012). Thus, the construction cost of extending the major vein and minor vein should be different. Also, the cell wall mass of veins should be considered when studying the scaling relationship between vein architecture and leaf size. These will be helpful for us to understand the covariant relationship of leaf venation structure with leaf size.

In addition, species with different vein types (palmate- vs. pinnate-veined) have different numbers of midribs, resulting in different major vein densities and tolerance to vein damages (Sack et al., 2008; Scoffoni et al., 2011). Species with different leaf habits (*i.e.*, evergreen versus deciduous species) also present significant differences in leaf size, leaf mass per area (LMA), vessel size, photosynthesis, and stress tolerance (Cavender-Bares and Holbrook, 2001; Wright et al., 2004; Yang et al., 2008). Therefore, leaf habits and leaf vein types might also change the general scaling relationship.

Here, we measured leaf size, leaf vein length, and leaf vein cell wall dry mass across 39 broad-leaved woody species within a subtropical forest community in Tiantong National Forest Park of China. These species belong to 27 genera in 18 families, including different leaf habits and leaf vein types, with leaf sizes ranging from 7.68 to 196.00 cm^2^. We aimed to (1) test whether the general scaling relationship of leaf vein density with leaf size holds across species within a community, (2) determine whether the ratio of major to minor vein density and vein cell wall mass per length are also related to leaf size, and (3) test whether the scaling exponents are consistent between different leaf habits and different leaf vein-type species. Additionally, we compiled the global dataset from Sack et al. (2012), redefined the major vein and minor vein as we did in this study, and reanalyzed and compared the scaling relationship between our data and data from Sack et al. (2012). This comprehensive study on the scaling trends of leaf venation with leaf size would provide a fundamental understanding of the adaptive significance of leaf size and venation and their ecological strategies.

## Materials and Methods

### Study Sites and Leaf Materials

Leaf materials were collected from 39 broad-leaved woody species (including 8 palmate-veined deciduous, 7 pinnate-veined deciduous, and 24 pinnate-veined evergreen species) in an evergreen broad-leaved forest of Tiantong National Forest Park, China (29°48′ N, 121°47′ E) on August 2019 when leaf expansion was completed. In all collected species, there were only a few that were not native species, but have been planted for many years in the park. The study site has a subtropical monsoon climate. The mean annual temperature and precipitation from 2012 to 2017 was approximately 16.6°C and 1824.4 mm, respectively. The meteorological data were from the Zhejiang Tiantong Forest Ecosystem National Observation and Research Station.

For each species, four to five healthy adult individuals within a similar environment were selected, and three to five random branches with tips at the outer edge of the middle layer of the plant crown were chosen. Healthy, undamaged, and fully developed current-year leaves located on the third or fourth leaf position were sampled for venation architecture and leaf traits measurements.

### Vein Systems Analysis

One leaf per branch from three branches on each of five individuals was collected. Therefore, a total of 15 leaves per species were used for vein system measurements. The leaf vein orders were classified and divided into major (including 1° and 2° veins) and minor veins (3° veins and higher-order veins) according to Ellis et al. (2009), Walls (2011). Firstly, cleaned fresh leaves were scanned using a scanner (LiDE 300, Canon, Vietnam), and leaf area and the length of 1° and 2° veins in each leaf were measured by ImageJ 18.0 (National Institutes of Health^1^). Secondly, all leaves were chemically cleared with 5% NaOH solution and boiled in a water bath with a constant temperature for 20–30 min. Then, 1-cm^2^-sized samples (avoiding 1° and 2° veins as much as possible) were cut from symmetrical locations of the tip, middle, and bottom of the leaves (6 samples per leaf, 15 leaves per species, see Supplementary Figure 1) and bleached in 5% NaClO solution, then stained with an alcoholic solution of toluidine blue (3%) overnight. The above protocols were similar to other studies (Scoffoni et al., 2011; Sack et al., 2012; Petruzzellis et al., 2019). Finally, three images of each sample (a total of 18 images per leaf) were obtained with a digital camera (Leica, DFC7000 T, Germany) mounted on an optical microscope (Leica, DM6B Wetzlar, Germany) at 40 × magnification. Then, minor vein length was measured using the phenoVein software with manual correction (Bühler et al., 2015). The major and minor vein densities were calculated by dividing leaf area with vein length in major and minor veins, respectively. The ratio of major to minor vein density was obtained by the major vein length divided by the minor vein length.

### Leaf Vein Cell Wall Mass Analysis

One leaf on a sun-exposed branch from each of the same five individuals measured for vein architecture was collected for leaf vein cell wall mass measurements (cell wall mass denotes cell wall dry mass except where we specifically mentioned fresh mass). After scanning and measuring the leaf area, the leaves were chemically cleared with the same protocols as described above for vein systems. Then, the leaves were washed with deionized water and dried with absorbent paper. Then, 1°, 2°, and minor veins were separated from the cleared leaves with scissors and tweezers before the fresh mass was measured with an analytical balance (±0.1 mg, Mettler Toledo, XPE 205, Switzerland). For the leaf vein cell wall extraction, we used the same protocol as Wang et al. (2017). Briefly, the weighted fresh vein was transferred to a centrifuge tube that was injected with 75% ethanol, and the samples were ground to homogenate with a grinder (70 Hz, 2 min). Then, the homogenates were washed into a 50 ml centrifuge tube with 75% ethanol and kept in an ice-cold bath for 20 min. Later, the homogenates were centrifuged at 1,000 RPM for 10 min, and sediments were washed according to a sequence of 1:7 (vein fresh mass/volume) of ice-cold acetone, methanol-chloroform mixture (1:1, V/V), and methanol. The supernatant in each washing was discarded, and the final deposit was dried in an oven at 70°C for 48 h. The dry mass of the powder is the vein cell wall mass, hence the cell wall mass of 1°, 2°, and minor veins were separately obtained. Except for vein traits mentioned above, the other current-year leaves located on the third or fourth leaf positions of selected branches were collected. Then, the leaf area and leaf mass were obtained for calculation of (LMA).

Ideally, the vein cell wall mass and vein length should be measured at the same leaf area to get the vein cell mass per vein length. However, we used different leaves with different leaf areas for these two measurements. Hence, the vein cell wall mass was converted proportionally to the corresponding vein cell wall mass in the leaf area that was used for measuring vein length for each branch in each species. Based on the LMA values, the same proportional conversion was performed for obtaining the leaf mass of the same leaf area that was used for vein length measurements. In this way, all traits were obtained with the same leaf area that was measured for vein length in each species. For ensuring that the vein cell wall mass conversion was correct, we tested the scaling relationship of vein cell wall mass with leaf area across species. Since the scaling exponents were not different between original vein cell wall mass measurements and converted data with the same leaf area in the vein length measurements, we confirmed that the conversion was correct. The leaf area of each species in the two measurements was similar. Particularly, the points were on the 1:1 line, and the slope did not deviate from 1.0 (*R*^2^ = 0.93).

### Data Analyses

Preliminary regression analyses showed that all bivariate relationships were log-log linear. Thus, log_10_-transformed data were used in all statistical analyses for scaling relationships. The bivariate relationship was described by the equation *y* = *ax^b^*, where *x* and *y* were two traits and *a* and *b* represented the intercept and slope of the linear relationship. We followed the other published papers on vein architecture and leaf trait studies in using ordinary least squares (OLS) or standard major axis (SMA) linear regression for fitting the data. They mainly depended on the calculation of the given trait and thus its relative level of measurement error (Niklas, 1994; Smith, 2009; Scoffoni et al., 2011; Price et al., 2012; Sack et al., 2012). The SMA is preferred for allometric scaling analyses, particularly when the measurement error in both variables is proportional and when there is no dependent variable (Warton et al., 2006). Furthermore, when measurement error in the ordinate variable is significantly higher than in the abscissa variable, the results analyzed by SMA are not as accurate as that in the OLS (Kimura, 1992; Sack et al., 2012). Therefore, for the scaling relationship of vein traits with leaf size, we used OLS, and alternatively, we used SMA. We noted that for most of the relationships, the directions of the relationships were not different regardless of using SMA or OLS, and the scaling exponents were similar when the correlation coefficients were high. The analysis of scaling relationships was conducted using Standardised Major Axis Tests and Routines (SMATR) (Falster et al., 2006), and confidence intervals for individual regression slopes were calculated following Pitman (1939). The common slops were obtained where homogeneity of slopes was demonstrated based on the methods of Warton and Weber (2002). Then, the differences in elevation of regression lines (*y*-intercept) were tested as in standard analysis of covariance (ANCOVA) (Wright and Westoby, 2002; Westoby and Wright, 2003; Yang et al., 2009).

Phylogenetically independent contrasts (PIC) were also performed for analyzing the correlation between functional traits throughout their phylogeny. The phylogenetic tree was constructed using PHYLOMATIC, which is based on the Angiosperm Phylogeny Group III classification of angiosperms (APG III^2^). The PIC analysis was conducted using the “ape” package (Paradis et al., 2004) in R 4.1.0 version.

To test trait differences between species groups of this study, the *t*-test was performed. The partial correlation analysis was performed for the intercorrelated relationship among the major vein density, the ratio of major to minor vein density, and leaf size across species, testing the relationship between two variables while holding the third variable constant (Scoffoni et al., 2011).

The global dataset (from Sack et al., 2012) was reanalyzed based on the same definition of major and minor veins as ours, so that the data are comparable. There were 485 species in the global dataset, including data from previous literature (36 species) and original data from their study, which had 410 of 449 species collected from the Daniel I. Axelrod cleared leaf image collection (Museum of Paleontology of the University of California, Berkeley, California). We extracted species in two ways. One contained detailed data of major and minor veins (63 species, called “Sack’s data”) to compare with our data. The other included all species from Axelrod, which accounted for most of the global dataset, including 1° and 2° but not more than 3° veins. Thus, we can get major vein data (401species) from Axelrod (called “Axelrod’s data”) to compare with ours or Sack’s data. In addition, because there was no significant difference in the *y*-intercept of major vein density and the ratio of major to minor vein density vs. leaf area between evergreen and deciduous species within pinnate-veined species in our results, only venation type species were separately analyzed when comparing with Sack’s data or Axelrod’s data.

## Results

### Relationships Between Leaf Vein Density and Leaf Size

In the current study, the leaf size ranged from 7.69 to 196.00 cm^2^, with a considerable range in total vein density from 52.04 to 122.54 cm^–1^. Detailed functional traits can be found in Supplementary Table 1.

**FIGURE 1:**
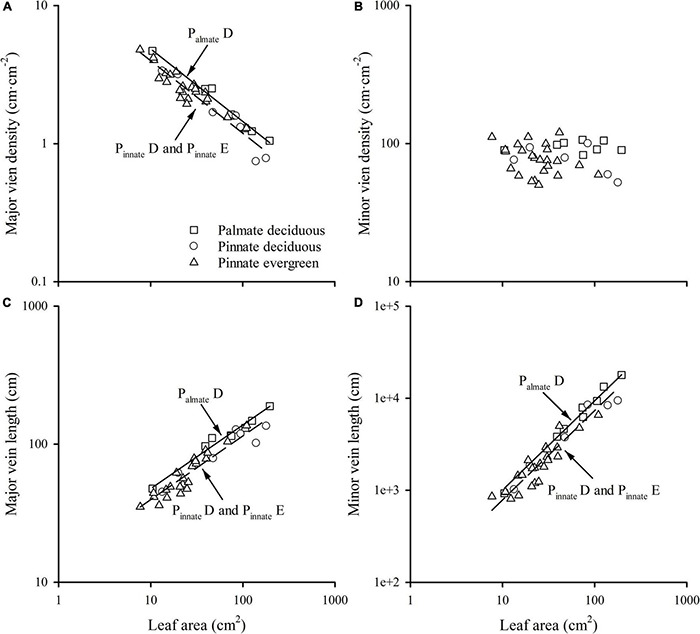
The relationships of the major vein density **(A)**, the minor vein density **(B)**, the major vein length **(C)**, and minor vein length **(D)** with leaf area across different group species. P_*almate*_D, plamate-veined deciduous species; P_*innate*_D, pinnate-veined deciduous species; and P_*innate*_E, pinnate-veined evergreen species.

A strong negative correlation between the major vein density and leaf area in palmate-veined deciduous, pinnate-veined deciduous, and pinnate-veined evergreen species groups was found (all *R*^2^ > 0.824), with a common slope of –0.525 [95% confidence interval (CI) –0.576, –0.450, *p* = 0.158], which did not differ significantly from –0.5 (Figure 1A and Table 1). The same result was found when leaf size was represented by lamina mass with a common slope of –0.453 (95% CI –0.561, –0.328, *p* = 0.517) (Table 1). These results indicated that the major vein density geometrically declined with leaf size. However, palmate-veined deciduous species had a significantly higher *y*-intercept than both the pinnate-veined deciduous and evergreen species (*p* < 0.01, Figure 1A and Table 1), suggesting that the palmate-veined species have a greater major vein density at the given leaf area than in the pinnate-veined species. However, deciduous and evergreen species showed no difference within the same pinnated-veined vein type. By contrast, the density of minor veins was independent of leaf size, as was the total vein density (Figure 1B and Table 1). The correlation between the major vein density and leaf size was also significant when expressed as correlated evolutionary divergences (Table 2).

**TABLE 1:** Parameters for the scaling relationships of vein traits and leaf traits.

*x*	*y*		*b*-*Value* (95% CIs)			*a*-*value*		
		Method	P_almate_ D	P_innate_ D	P_innate_ E	P_almate_ D	P_innate_ D	P_innate_ E
Leaf area	*Vein length*, cm							
(cm^2^)	Major vein	OLS	0.461(0.412, 0.545)			1.217a	1.134b	1.140b
			*R*^2^: 0.969	0.887	0.879			
	Minor vein	OLS	0.970(0.856, 1.053)			2.032a	1.926b	1.923b
			*R*^2^: 0.990	0.952	0.827			
	Total vein	OLS	0.958(0.846, 1.039)			2.064a	1.958b	1.955b
			*R*^2^: 0.990	0.952	0.830			
	*Vein density*, cm cm^–2^							
	Major vein	OLS	–0.525(–0.576, –0.450)			1.212a	1.122b	1.132b
			*R*^2^: 0.979	0.935	0.824			
	Minor vein	OLS	ns	ns	ns	–	–	–
			*R*^2^: 0.007	0.337	0.085			
	Total vein	OLS	ns	ns	ns	–	–	–
			*R*^2^: 0.003	0.387	0.103			
	*Vein cell wall mass per length*, g cm^–1^							
	Major vein	OLS	0.583(0.437, 0.721)			–4.968a	–4.702b	–4.614b
			*R*^2^: 0.672	0.887	0.554			
	Minor vein	OLS	ns	ns	ns	–	–	–
			*R*^2^: 0.001	0.390	0.021			
	Total vein	OLS	ns	ns	ns	–	–	–
			*R*^2^: 0.001	0.397	0.049			
	MVD/M_i_VD	OLS	–0.454(–0.554, –0.298)			–0.892a	–0.828a	–0.845a
			*R*^2^: 0.950	0.592	0.496			
Lamina mass	MVD	OLS	–0.453(–0.561, –0.328)			0.158a	0.010a	0.156a
(g)			*R*^2^: 0.764	0.734	0.635			
	MVD/M_i_VD	OLS	–0.391(–0.509, –0.230)			–1.802a	–1.825a	–1.688b
			*R*^2^: 0.682	0.843	0.376			
MVD	MVW/MVL	SMA	–1.295(–1.708, –1.048)			–3.575a	–3.422ab	–3.282b
			*R*^2^: 0.638	0.814	0.501			
M_i_VD	M_i_VW/M_i_VL	SMA	ns	ns	ns	–	–	–
			*R*^2^: 0.191	0.003	0.017			
MVD	MVD/M_i_VD	SMA	0.975 (0.820,1.130)			–1.967a	–1.865b	–1.868b
			*R*^2^: 0.965	0.872	0.377			
Leaf area	Lamina mass	SMA	0.960 (0.839,1.103)			–1.964ab	–2.100a	–1.880b
			*R*^2^: 0.721	0.862	0.872			

*CIs, confidence intervals; OLS, ordinary linear regression; SMA, standard major axis; P_almate_D, plamate-veined deciduous species; P_innate_D, pinnate-veined deciduous species; and P_innate_E, pinnate-veined evergreen species; MVD, major vein density, cm cm^–2^; M_i_VD, minor vein density, cm cm^–2^; MVL, major vein length, cm; M_i_VL, minor vein length, cm; MVD/M_i_VD, the ratio of major to minor vein density; MVM/MVL, the cell wall mass per vein length of the major vein, g cm^–1^; M_i_VM/M_i_VL, the cell wall mass per vein length of the minor vein, g cm^–1^. b-value is the common slope among different species groups by ordinary linar regression (OLS) or standard major axis (SMA) method; a-value is the y-intercept based on the common slope; the small letter after the a-value is the significance test, different letters mean the significant difference; ns represented the scaling relationship was not significant. R^2^ is the absolute coefficient of scaling relationship between two traits.*

**TABLE 2:** The regression slopes between functional traits (log-log transformed data) of correlated evolutionary divergences for 39 subtropical woody plants in Tiantong National Forest Park, China.

Traits (*x*-axis–*y*-axis)	Slopes	*R* ^2^	*P*-value
LA–MVD	–0.428	0.914	<0.001
LA–MVL	0.574	0.953	<0.001
LA–M_*i*_VL	0.994	0.960	<0.001
LA–MVM/MVL	0.433	0.638	<0.001
LA–MVD/M_*i*_VD	–0.427	0.835	<0.001
LM–MVD	–0.432	0.701	<0.001
LM–MVD/M_*i*_VD	–0.431	0.641	<0.001
MVD–MVW/MVL	–1.035	0.732	<0.001
MVD–MVD/M_*i*_VD	0.942	0.814	<0.001

*The OLS method on log-transformed variables was applied. All the regression lines were highly significant (p < 0.001). LA, leaf area; LM, lamina mass. For other abbreviations, see Table 1.*

Vein length was significantly and positively related to leaf area within each species group (all *R*^2^ > 0.827). The slope of vein length vs. leaf area was not different among species groups, with the common slope 0.461 (95% CI 0.412, 0.545, *p* = 0.072) and 0.970 (95% CI 0.856, 1.053, *p* = 0.202) for major and minor veins, respectively (Figures 1C,D and Table 1). The palmate-veined deciduous species were significantly greater in the vein length than two pinnate-veined group species at the same leaf area (*p* < 0.01, *y*-intercept in Figures 1C,D and Table 1), indicating that the major and minor vein lengths were both significantly larger in palmate-veined species than in pinnate-veined species. The result of the PIC analysis also showed a positive correlation between correlated evolutionary divergences (Table 2).

### Relationships Between Vein Cell Wall Mass per Length and Leaf Size

**FIGURE 2:**
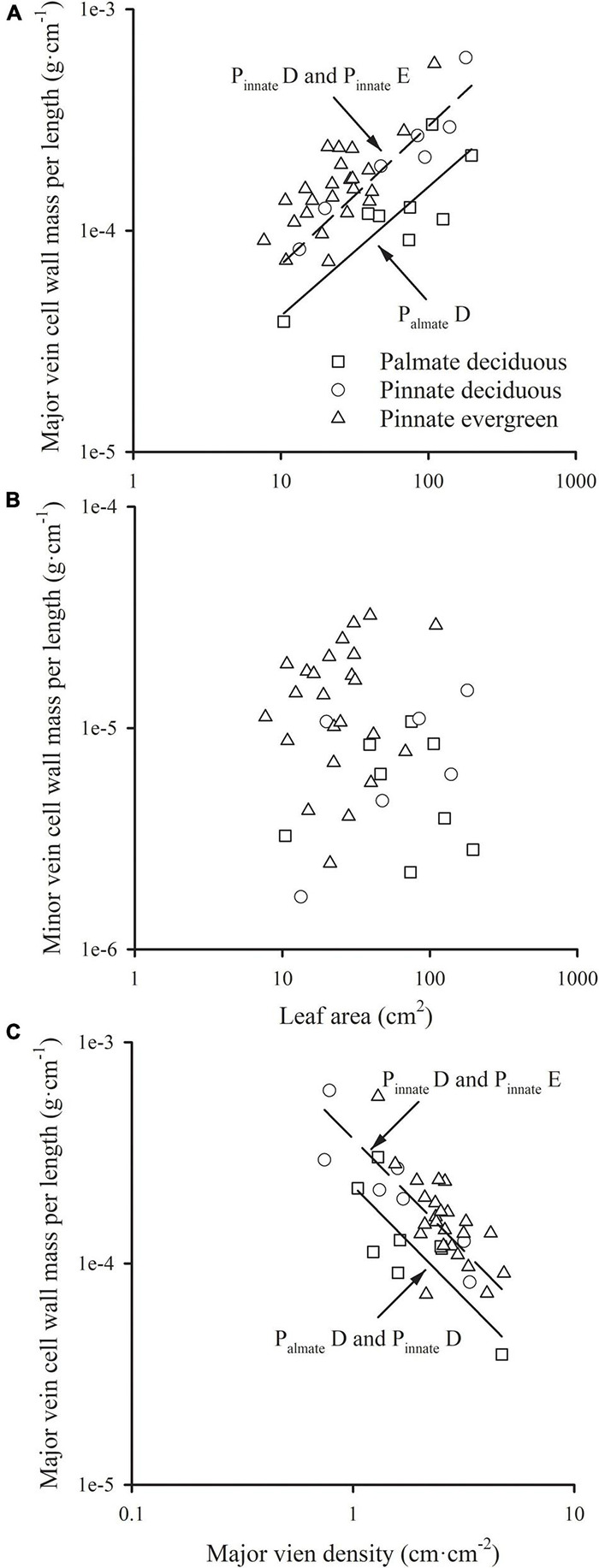
The relationships of the cell wall mass per length of the major vein **(A)**, and that of the minor vein **(B)** with leaf area; the relationship of the cell wall mass per length of the major vein with the major vein density **(C)**. For abbreviations, see Figure 1.

The major vein cell wall mass per length was significantly and positively related to leaf area in three groups (all *R*^2^ > 0.554), with a common slope of 0.583 (95% CI 0.437, 0.721, *p* = 0.919), which did not significantly deviate from 0.5 (Figure 2A and Table 1). This was consistent with the positive relationship between correlated evolutionary divergences (Table 2). The difference in *y*-intercept was found to be significant between palmate-veined deciduous species and pinnate-veined (evergreen and deciduous) species (*p* < 0.001), but not significant between two pinnated-veined species groups (*p* > 0.05, Table 1). In contrast, minor vein cell wall mass per length was independent of leaf size (Figure 2B and Table 1).

In addition, there was a significantly negative correlation between the major vein cell wall mass per vein length and the major vein density within each species group (all *R*^2^ > 0.501), with the common slope of –1.295 (95% CI –1.708, –1.048, *p* = 0.355), which marginally but significantly deviated from –1.0 (Figure 2C and Table 1). This suggested a tradeoff between the major vein density and the major vein cell wall biomass investment per unit vein length. The relationship was also strong when expressed as correlated evolutionary divergences (Table 2). However, there was no significant correlation between the minor vein cell wall mass per vein length and minor vein density in each species group (all *p* > 0.05).

### The Scaling Relationship of the Ratio of Major to Minor Vein Density With Leaf Size

**FIGURE 3:**
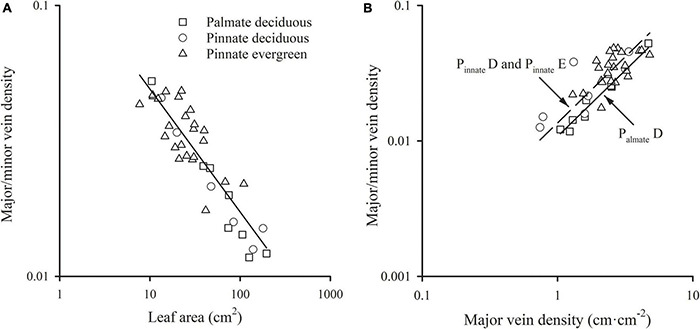
The relationships of the ratio of major to minor vein density with leaf area **(A)** and the major vein density **(B)**. For abbreviations, see Figure 1.

A significant scaling relationship was found between the ratio of major to minor vein density and leaf area within each species group (all *R*^2^ > 0.496). Neither the slopes nor the intercepts of the ratio of major to minor vein density vs. leaf area relationships differed among the species groups, with a common slope of –0.454 (95% CI –0.554, –0.298, *p* = 0.064) that did not significantly deviate from –0.5 (Figure 3A and Table 1). A similar scaling relationship was also found when the leaf size was represented by lamina mass (Table 1). These results indicated that the leaf vein density distribution was tightly correlated with leaf size. Furthermore, the ratio of major to minor vein density was significantly related to the major vein density in each species group, with the common scaling exponent of 0.975 (95% CI 0.820, 1.130, *p* = 0.472) not being different from 1.0. Thus, these two venation traits have an isometrical relationship (Figure 3B and Table 1).

In addition, the relationships between the ratio of major to minor vein density and leaf size, and between the ratio of major to minor vein density and major vein density were also highly significant when expressed as correlated evolutionary divergences (Table 2).

### The Comparisons Between Our Results and the Global Dataset

**FIGURE 4:**
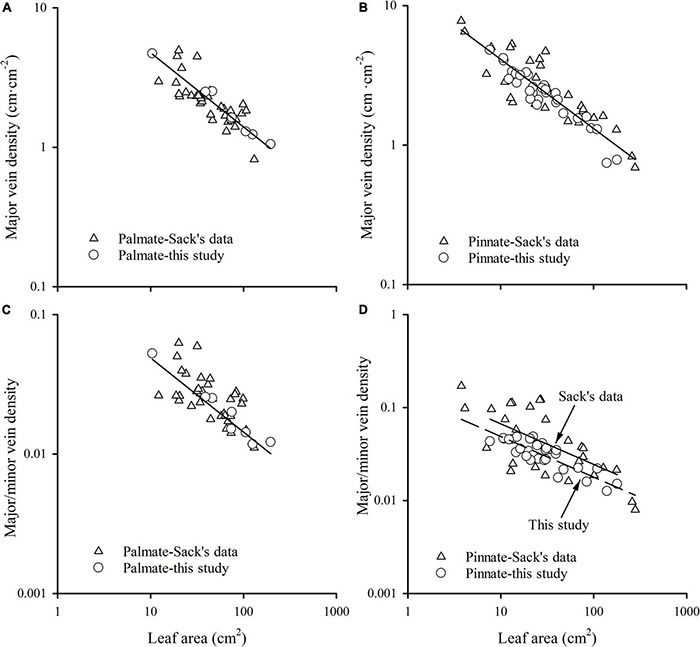
The relationships of major vein density of palmate-veined species **(A)**, major vein density of pinnate-veined species **(B)**, the ratio of major to minor vein density of palmate-veined species **(C)**, and the ratio of major to minor vein density of pinnate-veined species **(D)** with leaf area.

**FIGURE 5:**
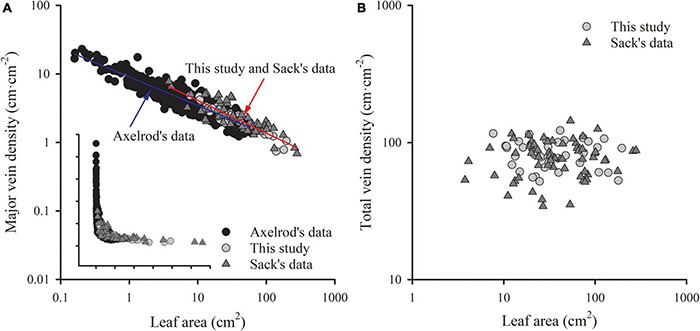
The comparisons of the scaling relationship of major vein density with leaf area **(A)**, and the independence of total vein density from leaf area **(B)** in our study (39 woody species within subtropical forestry community), Sack’s data (63 comprehensive species), and Axelrod’s data. The red line is the common slope of this study and Sack’s data, while the blue line is the slope of Axelrod’s data. Inset in panel **(A)** is the same plot but with raw data.

The major vein density negatively scaled to leaf area in both this study and Sack’s data (both *R*^2^ > 0.60). Neither the slopes nor the intercepts of relationships differed between these two groups, with common slopes of –0.532 (95% CI –0.594, –0.454) and –0.496 (95% CI –0.553, –0.439) for palmate- and pinnated-veined species, respectively, which did not significantly deviate from –0.5 (Figures 4A,B and Table 3). However, the scaling exponent in the Axelrod’s data (401 species) was significantly larger (less negative) than for this study and Sack’s data, with exponents of –0.408 (95% CI –0.440, –0.375) and –0.427 (95% CI –0.447, –0.407) for palmate- and pinnated-veined species, respectively, both being significantly deviated from –0.5 (Table 3). Consequently, when we plot the scaling relationship for Axelrod’s species, our data, and Sack’s data, there was no common slope among them in both venation type species (Figure 5A and Table 3).

**TABLE 3:** Parameters for the scaling of vein traits with leaf size analyzed by OLS method in this study, Sack’s data, and Axelrod’s data (Sack et al., 2012).

Vein type	Traits	Data source	N	*R* ^2^	*b*_1_-value	*b*_2_-value	*a*_2_-value	Tests for differences between groups
					Slope (95% CIs)	Common slope		
						(95% CIs)		*P*-value
Palmate	MVD	Sack’s	34	0.607	–0.478(–0.616, –0.339)	–0.532 (–0.594, –0.454)	1.196	*b*_2_: 0.434
	(cm cm^–2^)	This study	8	0.979	–0.544(–0.624, –0.464)		1.224	*a*_2_: 0.211
	MVD/M_*i*_VD	Sack’s	34	0.391	–0.402(–0.583, –0.221)	–0.509 (–0.602, –0.378)	–0.765	*b*_2_: 0.172
		This study	8	0.950	–0.545(–0.670, –0.420)		–0.792	*a*_2_: 0.382
	MVD	Axelrod’s	67	0.906	–0.408 (–0.440, –0.375)	–	–	*b*_2_: 0.016
		This study	8	0.979	–0.544 (–0.624, –0.464)			–
		Sack’s	34	0.607	–0.478 (–0.616, –0.339)			
Pinnate	MVD	Sack’s	29	0.740	–0.442(–0.545, –0.339)	–0.496 (–0.553, –0.439)	1.137	*b*_2_: 0.209
		This study	31	0.897	–0.519(–0.586, –0.453)		1.087	*a*_2_: 0.069
	MVD/M_*i*_VD	Sack’s	29	0.513	–0.509(–0.704, –0.313)	–0.437 (–0.523, –0.347)	–0.738	*b*_2_: 0.389
		This study	30	0.727	–0.415(–0.514, –0.317)		–0.876	*a*_2_: 0.006
	MVD	Axelrod’s	334	0.839	–0.427 (–0.447, –0.407)	–	–	*b*_2_: 0.042
		This study	31	0.897	–0.519 (–0.586, –0.453)			–
		Sack’s	29	0.740	–0.442 (–0.545, –0.339)			

*CIs, confidence intervals; OLS, ordinary linear regression; MVD, major vein density, cm cm^–2^; MVD/M_i_VD, the ratio of major to minor vein density. Parameters for the scaling of vein traits, that is, of b value in the equation log (trait) = a + b log (leaf size), across species in different data sources. b_1_-value is the slope of each data group, b_2_-value is the common slope of two or three groups. a_2_-value is the y-intercept based on the common slope. P-value is the test between groups for common slope b_2_-value and y-intercept a_2_-value, respectively.*

The ratio of major to minor vein density was significantly and negatively correlated with leaf area in both this study and Sack’s data (both *R*^2^ > 0.39), with common slopes of –0.509 (95% CI –0.602, –0.378) and –0.437 (95% CI –0.523, –0.347) for palmate- and pinnated-veined species, respectively, which did not differ from –0.5 (Figures 4C,D and Table 3). The *y*-intercept was also not different between this study and Sack’s data in the palmate-veined species. However, the Sack’s data had a significantly higher ratio of major to minor vein density than our data at a given leaf area in the pinnate-veined species. In addition, similar to Sack’s global dataset, our data showed a considerable range in total vein density. The two datasets, both used high-resolution images, did not differ significantly (Figure 5B, *p* = 0.231 of *t*-test, Supplementary Table 1).

## Discussion

Our results showed an extremely strong and consistent scaling relationship of leaf venation architecture with leaf size across species with different leaf habits (evergreen and deciduous) and leaf vein types (palmate-veined and pinnate-veined) within a community. The scaling relationship between leaf vein density and leaf size across species was the same as what was found in the global dataset (Sack et al., 2012). Additionally, we found that the leaf vein distribution (the ratio of major to minor vein density) and the major vein cell wall mass per length were significantly correlated with leaf size across species. Indeed, it is intriguing when different leaf habits and leaf vein types species have the same scaling relationships between leaf vein traits and leaf size. This pattern suggests that these leaf vein traits are of importance for leaf size and are governed by the law of physics or physiological requirements. These relationships are core discoveries in leaf structure and function, which have important ecological and biogeographic implications. These results also provide a new way to understand the optimization of leaf size.

### The Scaling Relationship of Leaf Vein Density With Leaf Size

We found a strong negative correlation of major vein density with leaf size across 39 woody broad-leaved species within a community. However, minor vein density was independent of leaf size (Figure 1). Since total vein density was mainly determined by the minor vein length per area, which accounted for > 97% of the total vein length in this study (Supplementary Table 1), the total vein density was also not related to leaf size. This was consistent with findings from the previous global-scale dataset and small groups with no more than 10 species in previous studies (Sack et al., 2008, 2012; Dunbar-Co et al., 2009; Scoffoni et al., 2011).

These relationships were robust across different leaf habits and leaf vein types, with the common slope of major vein density with leaf size not different from –0.5 (Figure 1 and Table 1). The consistent results when leaf size was represented with leaf area and lamina mass was because of the isometric relationship between leaf area and lamina mass (*b* = 0.96, 95% CI 0.84, 1.10, Table 1). This robust geometrical scaling of the major vein density with leaf size could be directly demonstrated from the scaling relationship between vein length and leaf area in the current study. The significant relationship between major vein length (*MVL*) and leaf area (*A*) among all groups showed a scaling slope not significantly different from 0.5 (*b* = 0.46; Figure 1C and Table 1), *i.e.*, *MVL* scaling with *A* was described as *MVL*∝ *A*^0.46^, and the leaf area scaled with leaf area as *A*∝*A*^1^. Therefore, the major vein density was determined by *MVD* = *MVL*/*A* ∝*A*^–0.54^, and the scaling exponent was not different from –0.5 (Figure 1 and Table 1). Additionally, considering the geometric dimensions, geometric scaling predictions have been derived (Niklas, 1994) by treating each fundamental trait as an area (*A*) as a two-dimensional variable and length (*L*) as a one-dimensional variable. Hence, vein density is an *L*/*A*∝*A*^–0.5^. Thus, we can say that the major vein density geometrically declined with leaf size. However, the scaling of minor vein length (*M_*i*_VL*) with leaf area was described as *M_*i*_VL*∝ *A*^0.97^, which was not different from 1.0 (Table 1). Thus, the minor vein density was determined by *M_*i*_VD* = *M_*i*_VL*/*A* ∝ *A*^–0.03^, suggesting that *M_*i*_VD* did not change with leaf size. These trends can also be explained by the development mechanism of venation according to the synthetic model (Sack et al., 2012). With the leaf development, the 1° and 2° veins are formed during a “slow” limited expansion phase due to cell proliferation, and the vein density peaks as procambium forms and declines as leaves are pushed apart during subsequent rapid expansion. Thus, the major vein density would geometrically decline with increasing leaf size. In contrast, the 3° and other higher-order veins are principally formed during a “rapid” dramatic expansion phase mainly because of cell expansion, although cell divisions continue. Thus, the minor vein density stabilizes as their initiation, and is maintained during leaf expansion (Sack et al., 2012). The declining trend of major vein density with leaf area was consistent among different leaf vein types and leaf habit groups. However, the palmate-veined species had higher major vein density than pinnate-veined species at a given leaf area (Figure 1A), which can be mainly ascribed from palmate-veined species having more midrib compared with pinnate-veined species (Sack et al., 2008; Scoffoni et al., 2011). Additionally, major vein length in palmate-veined species was significantly higher than in the pinnate-veined species at the same leaf area (Figure 1C and Table 1), which also contributed to the above difference. Even though evergreen species normally have smaller leaf area, photosynthesis efficiency, and higher LMA than for deciduous species (Zhang et al., 2013; Qi et al., 2021), major vein length and the major vein density were both insignificantly different between deciduous and evergreen species within the same leaf vein type in the current study (*p* > 0.05). Therefore, the *y*-intercept of scaling relationship in leaf vein density with leaf size was only impacted by vein type, not by leaf habit. Also, in the future, the evergreen and deciduous species could be combined within the same leaf vein type to study the scaling relationship of leaf venation architecture with leaf size.

The exponent of the geometrically scaling between major vein density and leaf area (no different from –0.5) in this study was different from that in the global dataset, which has the major vein density conservatively declined with leaf size (*b* = –0.341, 95% CI –0.360, –0.322) (Sack et al., 2012). This difference is not caused by different definitions of the major vein, but by the different range of leaf size of the studied species. When we reanalyzed the scaling relationship from the global dataset by recalculating major vein density based on the same definition of ours, the scaling exponent was still significantly larger than –0.5 (common slope of palmate- and pinnate-veined species, *b* = –0.405, 95% CI –0.383, –0.427), in agreement with original results reported in Sack et al. (2012). However, considering the data source, we found that the scaling exponent of Axelrod’s data (401 species) was significantly larger than –0.5 (Figure 5A and Table 3), while the scaling exponent of Sack’s data (63 species including major and minor veins) was not significantly different from –0.5, in agreement with our results (Figures 4A,B and Table 3). The conservative scaling exponent (>–0.5) found in the original global dataset was mainly determined by the Axelrod’s data because Axelrod’s species accounted for 85% of total species. The significant difference in scaling exponent between Axelrod’s data and ours or Sack’s data might attribute to the source of species collected by Axelrod or their venation treatment. We noted that the leaf area of the species collected by Axelrod ranged from 0.16 to 57.67 cm^2^, compared to 7.69 to 196.00 cm^2^ in our study and 3.77 to 279.65 cm^2^ in Sack’s 63 species. Thus, most species collected by Axelrod were smaller than ours or Sack’s, which could be related to the environment or climate condition. Therefore, we could say that within the same subtropical broad-leaved forest community, the major vein density was geometrically declined with leaf size, and this scaling exponent is consistent with another dataset that includes big leaves, but not with that including only small leaf species (Axelrod). The conserved scaling relationship between major vein density and leaf size may have important implications for understanding leaf size evolution, biogeography, physiological adaptation, and paleobiology. Further research work could be done to test whether the scaling exponent is related to the climate by studying different communities with different climate/environments. This will provide a new understanding of the species distribution based on the relationship between leaf venation structure and leaf size.

### The Scaling Relationships of the Vein Cell Wall Dry Mass per Length With Leaf Size and Vein Density

Despite that the total dry mass of the cell wall of veins increased with leaf size across species in this study (in each group, *R*^2^ > 0.50, *p* < 0.001), the cell wall mass per vein length was not fixed with the leaf growth, and the major and minor veins have different patterns. There was a significantly positive correlation between the cell wall mass per length and leaf size in major veins (Figure 2A and Table 1), but not in minor veins (Figure 2B and Table 1). As minor veins usually account for most of the total vein length, total vein cell wall mass per length was not significantly related to leaf size (Table 1). The above difference between major and minor veins could be caused by different diameter growth patterns of them during leaf development. The 1° and 2° veins have a prolonged diameter growth with the leaf development, and the power scaling exponent of vein diameter vs. leaf area was 0.452 (95% CI 0.426, 0.480) and 0.368 (95% CI 0.344, 0.394) for 1° and 2° veins, respectively (Sack et al., 2012). The thickening of major veins will lead to the increase of major vein volume per leaf area, resulting in the increase of cell wall biomass investment in the major vein (Niinemets et al., 2007; Niklas et al., 2007). However, minor veins (3° and higher-order) rapidly reach maximum diameter, and there is no correlation with leaf area (Sack et al., 2012). Hence, with the increase of leaf size, the cost of lengthening the major vein will increase, while the cost of that for the minor vein is relatively stable.

The results showed that the cell wall mass per unit length of major vein allometrically scaled with leaf area, with the scaling exponent not significantly different with 0.5 (Figure 2A and Table 1) across leaf habits and leaf vein types, indicating that the increase of cell wall mass per unit length of major vein could not keep up with the increase in leaf area. In other words, the leaf area that could be obtained by investing in the biomass of major vein cell wall per unit length increased with leaf area, *i.e.*, “increasing returns.” Based on this allometrically relationship, it will be beneficial for the plant to increase the cell wall mass per unit length of the major vein. However, it is impossible for plants to infinitely increase their investment in the major vein cell wall mass per length, because the major vein density would decline with the increase of cell wall mass per unit vein length of major veins (Figure 2C and Table 1). Therefore, there is a trade-off between the length growth and thickness growth of the major vein at a given biomass investment for the major vein. For a leaf, the greatest mechanical stress occurs along its longitudinal axis (Anita et al., 2001), and the mechanical reinforcement is determined by its low-order veins (Kull and Herbig, 1995). With the increase of leaf size, the larger major veins are required to provide stronger mechanical support, and increasing the diameter of the major vein increases the hydraulic conductivity within the leaf, which is the premises to improve the water transport efficiency of the whole plant (McKown et al., 2010). Consequently, at the same major vein cell wall mass investment, plants will prefer increasing vein diameter but not extending the vein length, leading to a tradeoff between the major vein cell wall mass per length and major vein density. This might be one of the reasons for the continuous increase in thickness of the major vein, while the major vein density peaks as procambium forms during leaf growth.

The trade-off of cell wall mass per unit length of the major vein and major vein density was of great significance for the optimization of leaf size. Although a thicker major vein is more conducive to support a larger leaf size, it will limit the length of the major vein and shorten the water transport distance within the major vein, which would impact the whole leaf hydraulic conductance. In order to maintain the water transport efficiency, it is impossible to increase leaf area by infinitely increasing the cell wall mass per unit length of the major vein. Rather, plants reach a reasonable leaf size due to this trade-off.

### The Ratio of Major to Minor Vein Density Scales With Leaf Size

In this study, the ratio of major to minor vein density was significantly scaled with leaf size among different species groups, which was consistent with a 10 species study (Scoffoni et al., 2011). This could be because the major and minor veins have different functions. For example, major veins (primary and secondary veins) act as the support and distribution network for leaves (Roth-Nebelsick et al., 2001; Ellis et al., 2009), while minor veins act as the sites of exchange between the mesophyll and the vascular system (Haritatos et al., 2000; Sack and Holbrook, 2006). Hence, the different distribution patterns of major and minor veins within a leaf would be preferred by different leaf sizes for adaption to the specific environment. In addition, this negative correlation could be ascribed to the slower speed of the increase in major vein length with leaf area compared to that of the minor vein. The scaling exponents were not far from 0.5 and 1.0 for major vein length and minor vein length, respectively (Figures 1C,D and Table 1). Thus, the ratio of major to minor vein density scaled with leaf area with an exponent not different from –0.5 (Figure 3 and Table 1). The *y*-intercept of this scaling relationship was not different between different leaf vein types or leaf habitats, although at the same leaf area major vein length was higher in palmate-veined species than in pinnate-veined species (Figure 2 and Table 1). However, the same trend was found in the minor vein length vs. leaf area. Therefore, the *y*-intercept difference was offset during the major vein length divided by the minor vein length to analyze the scaling relationship between the ratio of major to minor vein density and leaf area. The above results demonstrated that the scaling relationship between the ratio of major to minor vein density and leaf area was robust, approximately –0.5, regardless of palmate- or pinnate-veined species and evergreen or deciduous species.

The ratio of major to minor vein density was scaled with leaf area in our study and Sack’s data (63 species). This stable scaling relationship indicated that the ratio of major to minor vein density was another key venation trait linked with leaf area that maintained the same scaling exponent –0.5, which could be used for explaining the evolution of leaf size and adaptation to the environment. Also, the ratio of major to minor vein density was isometrically related to the major vein density [SMA results: *b* = 0.975 (95% CI 0.820, 1.130)] in our data (Table 1). However, within the subtropical community, we found that the evergreen species have higher mean values in the ratio of major to minor vein density and smaller leaf area compared to deciduous species within the same pinnate-veined species (Figure 3, both *p* < 0.05 by *t*-test). These trends are beneficial for species with small leaf areas because the higher the ratio of major to minor vein density, the smaller leaf area and more tolerance to leaf xylem cavitation (Scoffoni et al., 2011). This can be extended to the global scale species, of which small leaf species can survive unfavorable situations with a higher ratio of major to minor vein density.

The dramatic linkage between venation architecture (including major vein density and the ratio of major to minor vein density) and leaf size in the subtropical forest community or at the global scale (Figures 1, 3, 4) provides a hydraulic mechanism for explaining the ecological or biogeographical distribution of leaf size. Small leaves are predominant in drier and more exposed habitats (Givnish, 1987; Peppe et al., 2011; Sack et al., 2012), while large leaves in moister and/or shaded habitats (Givnish, 1987; Fonseca et al., 2000). A spatially explicit model showed that the greatest impact for the increase of *K*_leaf_ in the reticulate hierarchy system was from increases in major vein conductivity and in the minor vein density (McKown et al., 2010). Hence, the major veins normally have long and wide conduits (Choat et al., 2005), which in turn have higher vulnerability to cavitation (Choat et al., 2005; Blackman et al., 2010; Scoffoni et al., 2011). Because of this, higher major vein density in small leaves provides redundant hydraulic “superhighways,” *i.e.*, pathways around embolized major veins (Sack et al., 2008, 2012; Scoffoni et al., 2011). Both a high major vein density and a high ratio of major to minor density could reduce hydraulic vulnerability (Scoffoni et al., 2011). This study showed that small leaves generally have higher major vein density and ratio of major to minor vein density across leaf habits and leaf vein types (Figures 1, 3). Therefore, the hydraulic mechanism in providing the benefit of small leaves in dry and exposed habitats are the tight scaling relationships between leaf major vein density and leaf area, and between the ratio of major to minor vein density and leaf area. With a partial correlation analysis, the relationship of the ratio of major to minor vein density with leaf area was still significant after partialing out major vein density (*r* = –0.585, *p* < 0.01), and the correlation of major vein density with leaf area was also significant after partialing out the ratio of major to minor vein density (*r* = –0.778, *p* < 0.01) for pooled data of our study. These results indicated that the major vein density and the ratio of major to minor vein density both played a key role in leaf size distribution. Small leaves would have a lower hydraulic vulnerability that is preferred in dry habitats (Ackerly et al., 2002; Bragg and Westoby, 2002; McDonald et al., 2003; Sack et al., 2012). This provides an explanation for the fact that leaf size declines as annual temperature and precipitation decrease (McDonald et al., 2003).

In conclusion, we found strong correlations of the major vein density and the ratio of major to minor vein density with leaf size, and the isometrical relationship between the major vein density and the ratio of major to minor vein density across 39 species within a subtropical forest. These findings were confirmed by reanalyzing the global dataset. However, these relationships were not found in small leaf species collected by Axelrod, thereby asking for further studies to test this scaling exponent in different ecosystems with leaf size ranges. Interestingly, our results also demonstrated that these trends were robust in different vein types and leaf habits. However, palmate-veined species have higher major vein density and ratio of major to minor vein density at the given leaf size than pinnate-veined species, which was mainly due to a more uniform distribution of large veins in palmate-veined leaves (Niinemets et al., 2007). In contrast, evergreen and deciduous species have similar venation architecture at a certain leaf area. The linkages of the major vein density, the ratio of major to minor vein density with leaf size, and the negative relationship between the major vein density and the cell wall mass per vein length of major vein could have important implications in the optimization of leaf size, niche differentiation of coexisting species, plant drought tolerance, and species distribution.

## Data Availability Statement

The original contributions presented in the study are included in the article/Supplementary Material, further inquiries can be directed to the corresponding author/s.

## Author Contributions

DY and GP conceived the research plans, supervised the experiments, analyzed the data, and wrote the manuscript. GP performed most of the experiments. YX, MY, and XW performed the leaf vein length measurements. WZ and ZC performed the leaf vein cell wall mas measruments. Y-JZ helped to revise the manuscript. All authors contributed to the article and approved the submitted version.

## Conflict of Interest

The authors declare that the research was conducted in the absence of any commercial or financial relationships that could be construed as a potential conflict of interest.

## Publisher’s Note

All claims expressed in this article are solely those of the authors and do not necessarily represent those of their affiliated organizations, or those of the publisher, the editors and the reviewers. Any product that may be evaluated in this article, or claim that may be made by its manufacturer, is not guaranteed or endorsed by the publisher.

## Footnotes

1
https://imagej.nih.gov/ij/

2
http://phylodiversity.net/phylomatic/
