# Supplementary material for: Leaf Venation Architecture in Relation to Leaf Size Across Leaf Habits and Vein Types in Subtropical Woody Plants
Source: Front Plant Sci. 2022 May 6;13:873036. doi: 10.3389/fpls.2022.873036 (PMC9121095; doi:10.3389/fpls.2022.873036)
Supplement: Supplementary file 2 [file Image_1.pdf]

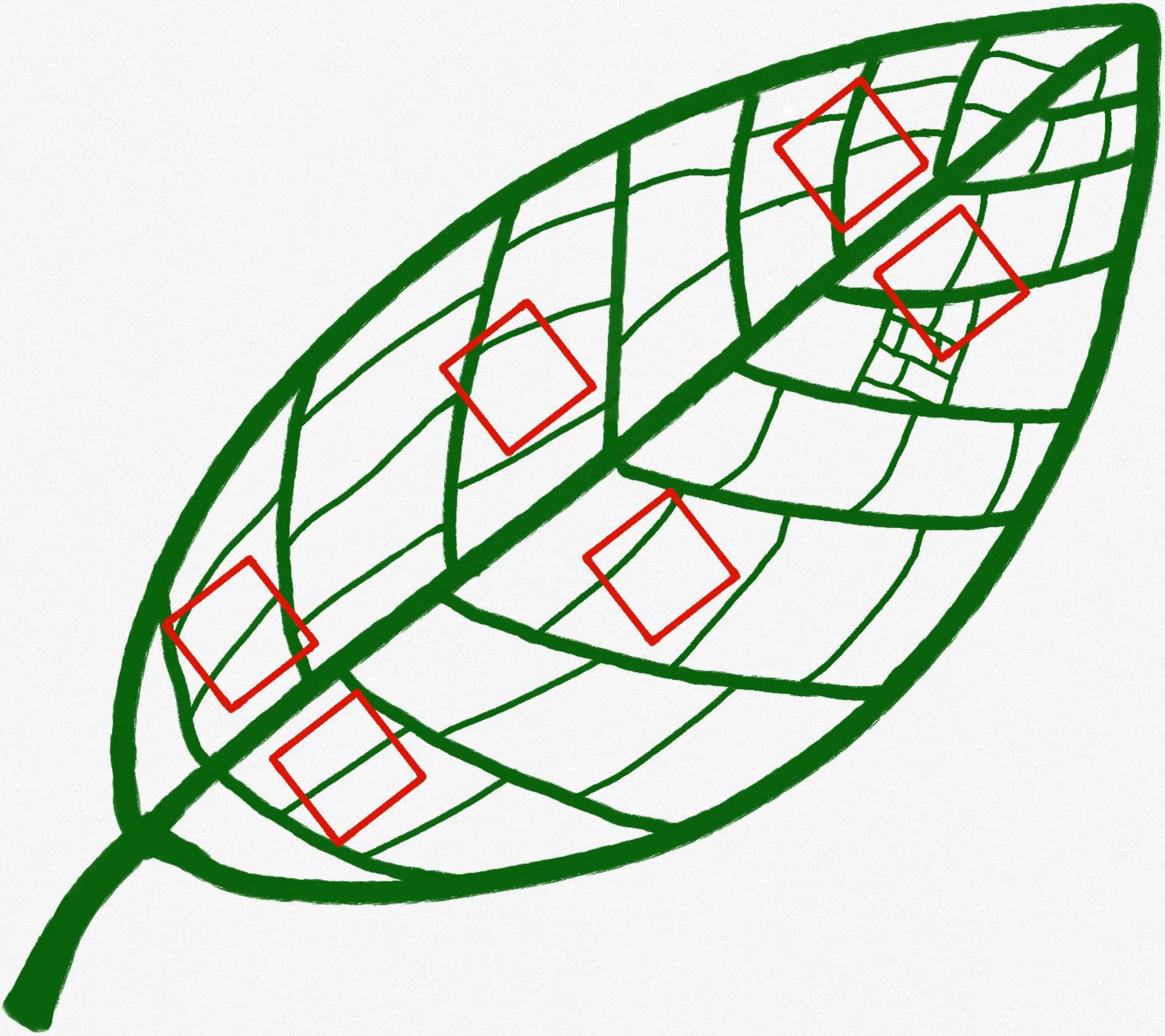

Figure S1: The illustration of sampling positions for minor veins within a leaf. Six subsamples were selected from symmetrical locations of the tip, middle, and bottom of the leaf.
